# Combinatorial constraint coding based on the EORS algorithm in DNA storage

**DOI:** 10.1371/journal.pone.0255376

**Published:** 2021-07-29

**Authors:** Li Xiaoru, Guo Ling

**Affiliations:** 1 Hulunbeier Vocational and Technical College, Hulunbeier, Inner Mongolia, China; 2 Baidu Co., Ltd., Shanghai, China; Polytechnical Universidad de Madrid, SPAIN

## Abstract

The development of information technology has produced massive amounts of data, which has brought severe challenges to information storage. Traditional electronic storage media cannot keep up with the ever-increasing demand for data storage, but in its place DNA has emerged as a feasible storage medium with high density, large storage capacity and strong durability. In DNA data storage, many different approaches can be used to encode data into codewords. DNA coding is a key step in DNA storage and can directly affect storage performance and data integrity. However, since errors are prone to occur in DNA synthesis and sequencing, and non-specific hybridization is prone to occur in the solution, how to effectively encode DNA has become an urgent problem to be solved. In this article, we propose a DNA storage coding method based on the equilibrium optimization random search (EORS) algorithm, which meets the Hamming distance, GC content and no-runlength constraints and can reduce the error rate in storage. Simulation experiments have shown that the size of the DNA storage code set constructed by the EORS algorithm that meets the combination constraints has increased by an average of 11% compared with previous work. The increase in the code set means that shorter DNA chains can be used to store more data.

## I. Introduction

IDC reports that the total amount of global information data will grow to 175 ZB in 2025. Mass data brings convenience to people but is also accompanied by challenges. How to reasonably use and store massive amounts of data has become a difficult problem. Emerging technologies such as artificial intelligence and big data have solved the problem of using large data sets, but storage remains a challenge as it requires a high-density, high-stability storage medium. Deoxyribonucleotide (DNA) is recognized as a potential storage medium because it has a high density, high storage capacity, strong durability, long life and low energy consumption. The theoretical storage density of DNA, composed of base ATGC, is twice that of traditional binary storage. DNA data storage technology, an organic combination of biotechnology and information technology, is a new type of storage technology of great significance for promoting low-energy storage and big data storage.

One of the earliest applications of DNA storage was in 1988, when Joe Davis [1] and researchers at Harvard University stored a Germanic picture representing female life and the Earth in the DNA sequence of *Escherichia coli* and showed that it could be restored by decoding. In recent years, DNA storage technology has gradually become a research hotspot around the world. At the beginning of this century, Bancroft et al. [2] proposed a simple method of codon triplet encoding, demonstrating the great potential of DNA as a storage medium. Erlich’s team used a DNA fountain coding method to store a complete 2.14 × 106-byte computer operating system, movies and other files and used Illumina sequencing to retrieve information [3]. Chen [4] and others introduced a method of using silica balls to protect DNA storage information. The DNA load of the DNA storage using silica balls increased to 3.4 wt%. Compared with the previous DNA storage method, the silica material has superior performance in DNA storage density and stability. Carbon nanotubes (CNTs) are a new type of composite material, used by Zhang et al. [5] to improve DNA storage performance. They condensed DNA strands on the surface of one-dimensional CNTs and developed a new type of tubular nucleic acid (TNA). Atomic force microscopy (AFM) imaging shows that TNAs present a unique pattern with characteristic heights and distances that can be used for two-dimensional encoding of CNTs. In the quantitative analysis of the DNA storage process, Wang et al. [6] used the most advanced droplet digital polymerase chain reaction (ddPCR) technology to monitor the long-term storage of DNA. Their experiments proved that ddPCR is an effective method for detecting DNA storage. In addition, Grass et al. [7] reported a strategy of using personal genetic information to securely store valuable information in synthetic DNA based on security considerations as well as using personal genetic information to personalize key protection. In 2020, Meiser [8] released a DNA storage experiment program, providing steps and precise instructions for converting digital information into DNA sequences and then regaining the information through DNA sequencing. Chen et al. [9] proposed a DNA hard disk drive (DNA-HD) as a rewritable molecular storage system with DNA as the medium, and the data in the system can only be read after the correct key is provided, which can ensure the security of data storage.

The basic process of DNA data storage includes encoding information into DNA sequences, synthesizing the required DNA sequences, storing these DNA sequences to form a database and potentially storing the database for a long time, reading the DNA sequence when it needs to be read, and then decoding the sequence into digital information according to the coding rules. One of the two most important processes in DNA storage is DNA coding. The purpose of DNA coding is to design efficient and stable DNA codewords. The code that meets all the requirements of a particular application can not only increase the storage density and ensure the integrity of the data but also reduce the error rate in the DNA storage process. Huffman coding is a mainstream coding method, often used in the field of information theory. In 2013, Goldman et al. [10] used Huffman coding in the DNA coding scheme, which effectively increased the coding potential to 1.58 bits/nt. After that, Bornholt et al. [11] improved Goldman’s coding scheme with the exclusive OR coding principle, using XOR operation to generate redundancy so that the third sequence can be restored for any two sequences. Song et al. [12] proposed a coding method that converts a 0–1 sequence into a DNA sequence and described a simple and effective coding technology implementation at a rate of 1.9 bits/nt, which achieved a lower coding rate. Immink [13] converted the standard binary maximum run length limited sequence to the maximum run length limited q-ary sequence, avoided long homopolymers and constructed a bit rate close to the theoretical maximum. Yazdi [14] and others used the WMU code to design a DNA storage code, and meet the WMU DNA sequence, but also need a large Hamming distance between each other, a balanced GC composition, and avoid primer-dimer by-products. Organick et al. [15] proposed an end-to-end DNA data storage system that demonstrated the ability of large-scale random access and the ability to correct errors caused by insertions and deletions. Song et al. [16] proposed a new three-base block coding scheme (SED3B) for reliable and orthogonal information coding of DNA in living cells. SED3B utilizes the inherent redundancy of DNA molecules and performs effective error correction by adding error detection bases in small data blocks. And through an error-prone PCR experiment on *E*. *coli* cells, it was confirmed that a 19% error rate can be corrected. Wang et al. [17] proposed a new type of content balance run length limitation (C-RLL), which has an efficient coding construction method and can simultaneously generate short DNA sequences that meet the maximum homopolymer run limitation and the balance GC content limitation. Lee et al. [18] introduced a new synthesis strategy for DNA data storage, which uses a template-independent terminal deoxynucleotidyl transferase (TdT) for synthesis under motion control conditions. This strategy synthesizes a DNA strand containing 144 bits and demonstrates the flow-type nanopore sequencing search, including addressing, which provides a powerful solution and theoretical basis for the development of DNA digital information storage technology. Fei et al. [19] designed a turbo-like decoder for a binary LDPC code DNA storage channel. Simulation results show that the bit error rates of binary LDPC codes are similar, but the speed is four times that of quaternary codes. OligoArchive, a DNA version of a relational database similar to the SQL relational database, was proposed by Appuswamy et al. [20]. OligoArchive is a DNA-based storage system with a relational database archiving layer architecture. The authors proved that OligoArchive can be implemented in experiments by building archive and recovery tools, and even SQL query statements can be used on OligoArchive. Yehezkeally et al. [21] considered the noise completely introduced by uniformly repeated sequences and the relationship between the Manhattan metric and equal-weight integer numbers. By using hyperplanes to define the intersection points across multiple walls, the existence of full-rate reconstruction codes was proved, and a method for constructing reconstruction codes was given. A storage model in which a data set is represented by M unordered sequences was proposed by Lenz et al. [22]. The errors in this model are the loss of the entire sequence and the point errors within the sequence, such as insertion, deletion and replacement. In this storage model, the Gilbert-Varshamov lower bound and the spherical packing upper bound of the error correction code up to the base are derived. In addition, Cao et al. [23] proposed a BMVO algorithm based on MFE constraints to construct a DNA storage code set and used the improved BMVO algorithm to construct a high-quality DNA storage code set. In the era of data proliferation, because the rate of data generation far exceeds the rate of increase of the storage density of hard disks, tapes and other media, researchers have begun to study new architectures and media types to store “unpopular” data that are not frequently accessed at a very low cost.

In this paper, an equilibrium optimization random search (EORS) algorithm is proposed to construct DNA storage codes that meet constraints in DNA storage. The code satisfies the Hamming distance constraint, GC content constraint and no-runlength constraint, has certain error correction capabilities, has high robustness characteristics, reduces coding complexity, shortens coding time and improves DNA storage system performance.

## II.  Coding constraints

### A.  Hamming distance constraint

Hamming distance is often used in coding theory to measure the similarity of two codewords. The smaller the Hamming distance [24,25] is in DNA coding, the greater the number of identical bases between two DNA code words, and the greater the possibility of non-specific hybridization. For different DNA sequences *a*, *b*, H(*a*, *b*) represents the number of different bases at the *i*-th position of the sequence *a*, *b*. The expression of the Hamming distance constraint is as follows, H(*a*,*b*) ≥ *d*, and the calculation formula of the Hamming distance is as follows:

$$H(a,b)=\sum_{i=1}^{n}h(a_{i},b_{i}),h(a_{i},b_{i})=\{\begin{array}{l} 0,a_{i}=b_{i}\\ 1,a_{i}\neq b_{i} \end{array}$$ (1)

### B.  GC content constraints

GC content refers to the ratio of bases G and C to the total number of bases in a DNA sequence. GC content [26] is a key indicator in DNA synthesis and sequencing, as GC content is closely related to the stability of a DNA sequence. It also has a very close relationship with melting temperature [12]. In order to follow the above rules, the designed DNA code should not have too much deviation in GC content. In this article, the GC content of a DNA sequence of length s is denoted as GC(s), and *|G+C|* represents the total number of G and C. In this study, *|G+C|* is assigned the value ⌊*s*/2⌋.


$$GC(x)=\frac{\left|G|+|C\right|}{\left|x\right|}$$ (2)

### C.  No-runlength constraint

The no-runlength constraint [27] requires that the DNA codeword should not include repetitive bases. Running the same nucleotide for a long time can cause DNA coding errors. For example, in TCCCCAC, C is repeated, so in synthesis and sequencing, it is easy to read a long C into a short C, which causes the error rate of DNA storage information to increase and reduces the read and write coverage rate. For codeword *d* (*D*_*1*_, *D*_*2*_, *D*_*3*_…*D*_*n*_), the length is *n* for any *i*:

$$D_{i}\neq D_{i+1}\;,i\in [1,n-1]$$ (3)

## III.  Equilibrium optimization random search

### A. Equilibrium optimizer

The equilibrium optimizer (EO) algorithm is inspired by a control volume in a hybrid dynamic mass balance, where the mass balance equation is used to describe the concentration of unreactive substances in a control volume weight dynamic balance. The mass balance equation provides a fundamental physical explanation for controlling the conservation of mass entering, leaving and produced within a volume. For more details, see the original paper [28].

The mathematical model of the EO algorithm is composed of the following three parts:

#### Step 1: Initialization

The initial concentration is constructed according to the number and dimension of uniformly and randomly initialized particles in the search space, and the formula is as follows:

$${\vec{v}}_{i}=c_{\min}+(c_{\max}-c_{\min})*r1\;i=0,1,2,\ldots ,n$$ (4)

Here ${\vec{v}}_{i}$ represents the concentration vector of particle *i*, *c*_max_,*c*_min_ respectively represent the upper and lower bounds of the dimension, and *r*_*1*_ represents a random vector between [0,1] and contains *n* groups of particles.

#### Step 2: Equilibrium pool and candidates

For all heuristic algorithms, there is an optimization objective based on their properties. For example, the ant colony algorithm [29] searches for ants’ food, the Wolf colony algorithm [30] searches for the prey of wolves and the EO algorithm searches for the equilibrium state of a system. However, during the optimization process, the EO algorithm does not know the concentration level to reach the equilibrium state, so it manually allocates the four best particles found in the equilibrium state, plus another particle containing the average value of the four best particles. These five particles help EO algorithms perform better in exploration and exploitation, and they all exist in an equilibrium pool:

$${\vec{p}}_{\mathrm{eq},\mathrm{pool}}=[{\vec{p}}_{\mathrm{eq}(1)},{\vec{p}}_{\mathrm{eq}(2)},{\vec{p}}_{\mathrm{eq}(3)},{\vec{p}}_{\mathrm{eq}(4)},{\vec{p}}_{\mathrm{eq}(\mathrm{avg})}]$$ (5)

#### Step 3: Update method of concentration

Using F helps the EO algorithm find a reasonable balance between exploitation ability and exploration ability. In a control volume, where the turnover can vary over time, suppose *λ* is a random vector between 0 and 1.

$$\vec{F}=e^{-\vec{\lambda }(t-t_{0})}$$ (6)

where *t* is the increment with iteration, for which the formula is shown as follows:

$$t={\left(1-\frac{iter}{t_{\max}}\right)}^{\left(a_{2}*(\frac{iter}{t_{\max}})\right)}$$ (7)

In (7), *Iter* and *t*_max_, respectively, represent the current number of iterations and the maximum number of iterations, and *α*_*2*_ is the fixed value to control the development capacity. In addition, parameter *α*_*1*_ is used to enhance the diversity and exploration capability of the population, as follows:

$${\vec{t}}_{0}=\frac{1}{\vec{\lambda }}\ln(-a_{1}\mathrm{sign}(\vec{r}-0.5)[1-e^{-\vec{\lambda }t})+t$$ (8)

The production rate (R) is another parameter used to improve the exploitation operator, which has the following formula:

$$\vec{R}={\vec{R}}_{0}*e^{-\vec{\lambda }*(t-t_{0})}$$ (9)

$${\vec{R}}_{0}=\vec{\mathrm{RCP}}*(\vec{c_{\mathrm{eq}}}-\vec{\lambda }*\vec{C})$$ (10)

$$\vec{\mathrm{RCP}}=\{\begin{array}{ll} 0.5r_{1} & r_{2}>\mathrm{RP}\\ 0 & \mathrm{otherwise} \end{array}$$ (11)

where $\vec{\mathrm{RCP}}$ is a random vector between [0, 1], *r*_*1*_ and *r*_*2*_ are both random numbers between 0 and 1 and $\vec{\mathrm{RCP}}$ is the control parameter of the generation rate, which ultimately determines whether the generation rate will be applied to the update process of the EO algorithm.

Finally, the updating equation of EO is as follows:

$$\vec{C}=\vec{c_{\mathrm{eq}}}+(\vec{C}-\vec{c_{\mathrm{eq}}})*\vec{F}+\frac{\vec{R}}{\vec{\lambda }*V}*(1-\vec{F})$$ (12)

Here *V* is assigned to 1, and *RP* is assigned to 0.5. For a more detailed introduction to the EO algorithm, please refer to Faramarzi’s paper [28].

### B. EORS algorithm

Although the EO algorithm uses parameters such as *α*_*1*_ to enhance the exploration ability of the population, the population richness of the EO algorithm still decreases in the later iteration, which greatly increases the probability of falling into a local optimum, especially when solving practical problems. For this reason, another mainstream search algorithm was added to the DNA code. The random search algorithm is a simple search algorithm suitable for large-scale data, although it is slow to calculate accurate results. Random search (RS) is a series of numerical optimization methods that do not need the gradient of the optimization problem, so RS can be used for discontinuous or differentiable functions.

RS works by iteratively moving to better positions in the search space sampled from the hypersphere around the current position. In this paper, the random search algorithm is used to process the output *S*_*EO*_ of the EO algorithm. The RS algorithm can expand the search scope of the EO algorithm and obtain a larger coding set. By initializing set *S*, all codes in set *S* and *S*_*EO*_ are judged one by one as to whether they satisfy combinatorial constraints. The pseudocode is shown in Algorithm 1.

**Algorithm 1. The pseudocode of updating the encoding set *S***.

*Input S*_*EO*_

*While* when the termination condition is not met

    Initializes the code set *S*

    *for s* in *S*

        *if* coding *s* in the coding set *S* and *S*_*EO*_ satisfy the constraint conditions

            Coding *s* joins coding set *S*_*EO*_;

        *end if* coding in coding set *S*_*EO*_ and *s* do not meet the constraint conditions

          I*f* don’t satisfy the code number is equal to 1

          Delete the codes in *S*_*EO*_ that do not meet the constraints and add the code *s* to *S*_*EO*_

        else

            Code s does not join the code set S_EO_

        *end if*

        *end if*

    *end for*

*end while*

*Output* the *S*_*BS*_

**Fig 1:**
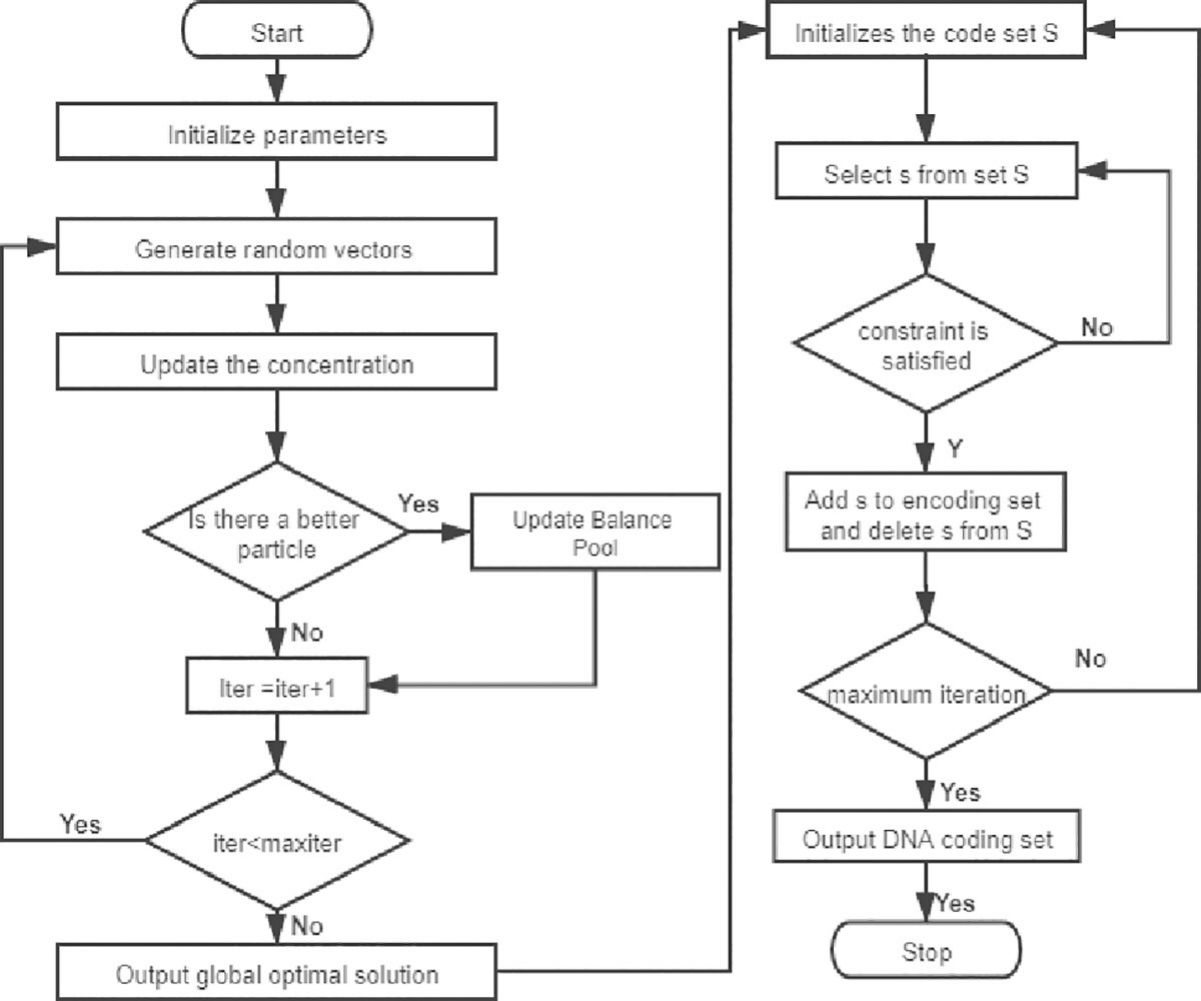
EORS algorithm constraint coding flowchart.

In the process of DNA constraint coding using EORS algorithm, the sum of Hamming distance is used as the fitness function.


$$fitness=\sum_{i=1}^{n}H(s,S_{i})$$ (13)

where *S* is the code set that has satisfied the constraint condition, *s* is the code word to be calculated and *n* is the set size of *S*. Correspondingly, the EORS algorithm constraint coding flowchart is given in Fig 1.

### C. Experimental environment

An Intel Core i5 computer with 4G memory and MATLAB 2016a software were used in the experiment. The results are shown in Tables 1 and 2. In order to facilitate the calculation, the numbers 0 to 3 were used to represent the four bases (*T→0*, *C→1*, *G→2*, *A→3*) in the DNA storage and coding process, and the parameters of the calculation process were consistent with the EO algorithm.

**Table 1:** The coding lower bound of A^*GC*,*NL*^(*n*,*d*,*w*).

*n*\*d*		3	4	5	6	7	8	9
4	Altruistic	11						
	EORS	12						
5	Altruistic	17	7					
	EORS	20	8					
6	Altruistic	44	16	6				
	EORS	55	21	8				
7	Altruistic	110	36	11	4			
	EORS	125	46	16	6			
8	Altruistic	289	86	29	9	4	4	
	EORS	364	110	38	15	5	4	
9	Altruistic	662	199	59	15	8	4	4
	EORS	737	226	71	26	11	5	4
10	Altruistic	1810	525	141	43	7	5	4
	EORS	1856	546	153	53	22	9	5

**Table 2:** DNA storage coding word when *n* = 9 and *d* = 6.

*A T C T G C T C A*	*A T C G A G A T G*
*G T A G T C G A T*	*T A T C G T A G C*
*T A G C T A G C T*	*T G T C A G C T A*
*G A C T A T C G A*	*A G T A C G T A C*
*A C A C A G T C T*	*C A T A T G A C G*
*G A T G T A C T C*	*G A T C A C T A G*
*T A G A C T C T G*	*T C G T C A T G A*
*A G A G C A G T A*	*A T G A T G C G A*
*C T A T G A C G T*	*A G A T A C A G C*
*T C A C T C A T G*	*T G C A T C T G T*
*T C T A G A G A G*	*G T A T C T A C G*
*C A C G C A T A T*	*T C A G A T C A C*
*C T G T A T G T C*	*C G C A G T A T A*

## IV.  Experimental results and analysis

In this paper, the DNA storage coding scheme based on constraint coding is simulated. EORS constructs the process of DNA storage coding set as follows.

*Step 1*: Generate the number of particles and initial concentration in the search space, and initialize the parameters required by the algorithm. Generate candidate sequences, which are derived from possible DNA codewords based on combination constraints.
*Step 2*: Initialize the DNA storage coding set. The Equilibrium pool and candidate pool strategies in the EO algorithm are used to sort the initial particle population, and the optimal fitness particle is selected. The optimal fitness particle is selected as the initial particle set.
*Step 3*: Balance exploitation and exploration capabilities through $\vec{F}$, and update the particles through (6)–(12).
*Step 4*: Use the updated results as the RS search input to make the next judgment on the DNA coding represented by the particles.
*Step 5*: Determine whether the constraint is satisfied. If the constraint is satisfied, add the new DNA coding word to the DNA storage coding set.
*Step 6*: Complete the number of iterations and output the DNA storage coding set.

### A. Boundary of DNA storage coding

The DNA coding set with defined length *n* and Hamming distance *d* that satisfies the Hamming distance constraint, GC content constraint and no-runlength constraint is A^*GC*,*NL*^(*n*,*d*,*w*). In Table 1, the result in the table is the lower bound of 4 ≤ *n* ≤ 10, 3 ≤ *d* ≤ *n* satisfying the constraint. In previous work, the value of *n* is generally less than 13, and *d* ≤ *n*. In this work, only the case of 4 ≤ *n* ≤ 10 was compared, because the time taken to construct the DNA storage coding set increased exponentially with the increase of *n*, and a sufficiently large DNA coding set was obtained when *n* = 10. The length of the DNA coding sequence is shown in Table 1, and the results obtained based on the EORS algorithm are listed and compared with the results of Limbachiya [27]. Limbachiya’s work was published in IEEE Communications Letters, the top journal in the field of communication coding. The underlined part indicates where EORS had better results under the same constraints. Altruistic represents Limbachiya’s coding scheme, and EORS is our algorithm. The results in the table show that the lower bound of the encoding set constructed by EORS has achieved ideal results in most cases. For example, when *n* = 8 and *d* = 3, the lower bound of the coding set constructed by the EORS algorithm is 26% higher than the previous result. This is because the EORS algorithm uses Generation probability and Equilibrium pool mechanisms to better balance the process of exploration and exploitation, and it adds a random search algorithm to further expand the candidate solution set at the later stage of the iteration. The results of the EO algorithm provide a good initialization for the random search algorithm, and the random search algorithm further expands the results of the EO algorithm. To be more convincing, a set of DNA stored code words satisfying the combinatorial constraint at *n* = 9 and *d* = 6 are presented in Table 2.

The lower bound of coding obtained by the EORS algorithm is better than that of the altruistic algorithm used by Limbachiya [27] in most cases. The altruistic algorithm is an intelligent algorithm that iteratively removes potential code words based on a greedy algorithm, which removes the “worst” candidate code words in each iteration. As the algorithm iterates, the altruistic algorithm greedily deletes the maximum number of code words in the radius range *d–1* until the code set has the minimum distance *d*. However, the altruistic algorithm based on the greedy algorithm does not consider the overall optimal solution, so it constructs only the local optimal solution in a certain sense. Therefore, the heuristic algorithm EORS is used in this work. EORS, based on the random search algorithm, is an improvement over the EO algorithm and has the advantages of fast convergence speed and high population richness.

### B. Coding rate

Using the EORS algorithm to build a larger DNA coding set under the same constraints provides a solid foundation for DNA storage in the next step and reduces the cost by more efficiently storing information. For a given length, finding a larger set of coding can not only reduce the cost but also increase the coding rate, where coding rate refers to the encoding rate. A coding rate of 1/4 means 1 code goes in and 4 codes come out. The greater the coding rate, the higher the efficiency. When the channel quality is relatively poor, more redundant information needs to be added to ensure that the receiving end can demodulate the signal correctly. More redundant information means a low coding rate. The minimum coding rate is a code that requires 3 redundant signals. The remaining code is 1/4 code. When the channel quality is good, a few redundant parity bits are needed to demodulate, and the coding rate can be increased. The system can select an appropriate coding rate according to the change of the channel so that users with good channel quality can get a higher rate and increase the average throughput rate. The coding rate is generally calculated by

$$R=\frac{\log_{4}M}{n}$$ (14)

where *M* is the size of the coding set and *n* is the length of the coding word.

**Fig 2:**
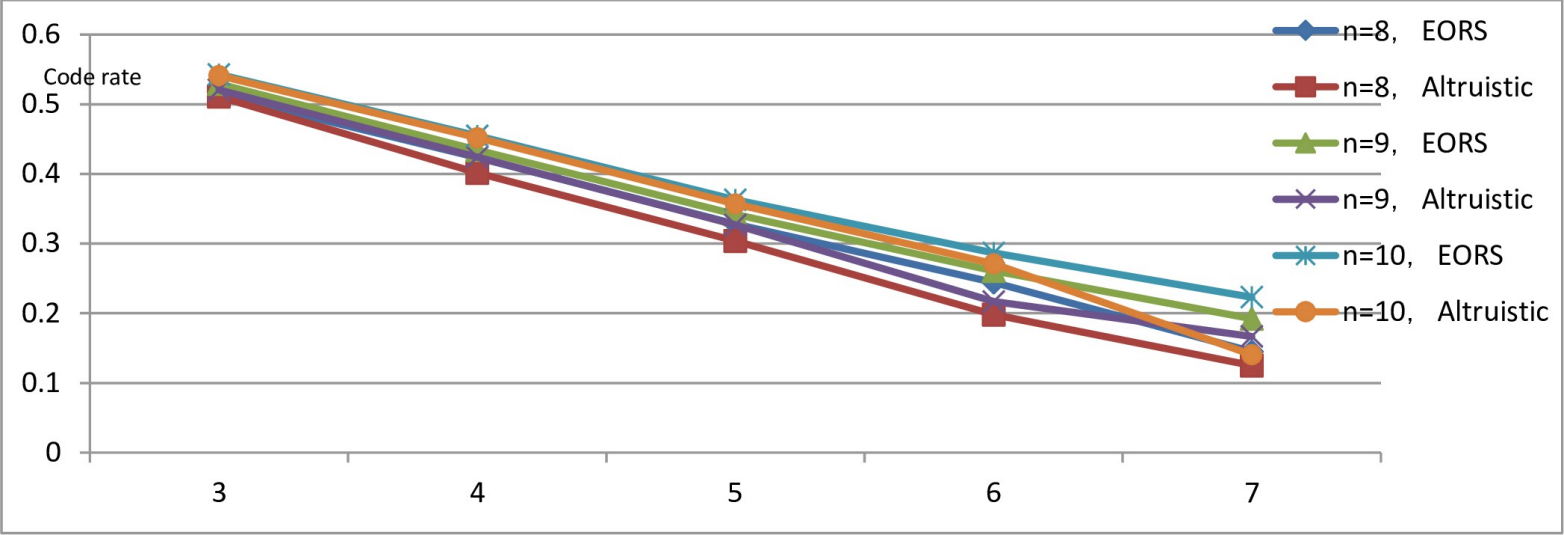
Coding rate of coding set constructed by EORS algorithm.

As shown in Fig 2, the figure lists the coding rate performance of the DNA storage code set obtained by the two encoding methods under *n* = 6, 7, 8, the *x*-axis represents the Hamming distance *d* and the *y*-axis represents the encoding rate. When the length of the codeword *n* is constant, the encoding rate constructed by the EORS algorithm is higher than that of the altruistic algorithm.

In particular, when *n* = 8 and *d* = 4, the coding rate of the EORS algorithm is R = log_4_ 110/8≈0.42, which is consistent with Limbachiya’s result when *n* = 9 and *d* = 3. The coding rate is the same, and the same performance can be achieved with a shorter code length. Therefore, the same performance can be achieved at a smaller code length. Moreover, the DNA storage coding constructed by EORS has better DNA storage capacity, coding rate and error correction ability. This result again demonstrates the superiority of the constrained coding scheme based on the EORS algorithm and its rationality in practical application. In addition, in other applications of DNA, such as long-time tracking [31] and neural network optimization [32], [33], DNA coding optimization [34,35], DNA molecular lock [36] and DNA image encryption [37], some excellent solutions have also emerged, providing new ideas for coding schemes in future work.

## V. Conclusion

In this paper, a DNA storage scheme based on the EORS algorithm is proposed to encode DNA storage through constraints. In this study, the combinatorial constraints of DNA storage coding problem are abstracted into a multi-objective optimization problem, and the approximate optimal solution of the DNA storage coding problem is determined by a heuristic algorithm. It not only takes full advantage of the heuristic algorithm’s ability to solve nonlinear multi-objective optimization problems but also applies the characteristic of low complexity of constraint coding to the field of DNA information storage, which improves the shortcomings of DNA being error-prone and exhibiting nonspecific hybridization in the synthesis and sequencing process. An effective DNA coding set is constructed by adding combinatorial constraints, and the results are obviously improved in comparison with previous work. Simulation experiments show that the coding scheme in this paper achieves ideal results in most cases, and the lower bound of the coding set is significantly improved, which further demonstrates the superiority of the EORS algorithm. The size of the DNA storage coding set is expanded by 11%–26%. Larger storage coding sets can store more effective information within the same DNA length, which not only reduces the cost but also improves the storage efficiency. Finally, this work compares the coding rate, which is an evaluation index of coding performance. As shown in Fig 2, with the same codeword length, the coding rate constructed by the EORS algorithm is higher than that constructed by the altruistic algorithm. The coding scheme based on EORS can achieve the same coding rate in a shorter sequence length. The same performance can be achieved in smaller coding lengths, increasing the efficiency and competitiveness of DNA storage systems.
